# On Pulmonary Consumption

**Published:** 1814-04

**Authors:** Richard Walker

**Affiliations:** Surgeon and Apothecary. Oxford


*For the Medical and Physical Journal.*

### *On Pulmonary Consumption; by* Mr. Richard Walker, of Oxford.

HAVING, through the medium of periodical publications of the first respectability, disposed of the philosophical portion of my various experiments and observations, I propose now to open my budget of *professional observations;* after having been actively and attentively employed in the professions of physic and surgery (the more immediate or direct objects of my pursuit) for nearly forty years, including the period at which my attention was first directed to those professions.

Nearly five and twenty years of this time were passed, during the earlier or rather middle period of life, at the Radcliffe Infirmary, where, all circumstances considered, I enjoyed singular opportunities of obtaining professional knowledge.

The professional observations alluded to, and which have been collected, and put together, in the course of three or four years, in order to be made public, consist entirely of the result of my own experience, founded upon observations, made upon patients who were immediately under my own care, and also upon those of others, which, conjointly, have furnished me with a degree of professional experience, rarely equalled, perhaps, in another individual.

The observations alluded to are intended ultimately for publication collectively; but since it is uncertain when they may be sufficiently arranged and completed for the fulfilment of that intention, I may request, through the medium of your valuable repository of professional information, the favor of your inserting certain portions of them which I may be induced to send, as opportunity shall offer.

The subject of the present paper, viz. *Pulmonary Consumption,* is unquestionably an important one; and doubtless any attempt, especially when founded upon matured experience, at pointing out the means of obviating such a complaint, or curing it when present, and, moreover, to show

 or or make apparent the fallacy and danger of relying upon the imaginary efficacy of *certain proposed specific medicines* in it, cannot but be acceptable to the faculty and the community.

In addition to my experience on other persons suffering under this destructive disease, or having a strong constitutional tendency to it, I have occasionally been compelled to exert my utmost efforts to avert it in myself; and I may venture to assert with confidence, that I have hitherto effected this by a rigid attention to the mode of treatment or management I have here laid down, and recommend for the adoption of others.

It is proper to observe, however, that this is not my first professional paper, two others being already before the public, viz. "Observations on the remarkable Efficacy of *Carrots*, under a new mode of Application, in the Cure of Ulcers and Sores;" and a paper "on the Treatment of *Burns* and *Scalds.*"

The observations already before the public, and such as may follow, are the result of deliberate and mature consideration; but from the pressure of various avocations, I have been obliged to content myself with the hope, respecting the composition itself, that it merely conveys my sentiments intelligibly; and although some of my observations may appear somewhat trifling or insignificant in detail, they will be found important in practice.

In the course of any professional observations I may have occasion to present in future, as well as in the present instance, particular care will be taken to deliver my sentiments and opinions decisively and unequivocally, at the same time avoiding as much as possible, without sacrificing that intention, giving offence to any one. It will be likewise my endeavour to remove every difficulty, and to obviate every objection, which might be started by others against the validity of some of my opinions, which may differ, perhaps, from those ordinarily received at this time, or any injurious tendency they might be supposed to have.

My object and wish is, that the community should be benefitted by my experience; and I am confident, that by pursuing the rules I have laid down in the present instance, and likewise in those which may follow, such will be the necessary consequence.

It will be apparent that the following paper is a portion only, or a detached part, of a general work: due allowance will therefore be made for any references or relations it may appear to have to other parts of that work.

The preceding observations, I presume, may be considered dered not unnecessary, or superfluous, at the commencement of my plan of intended professional communications.

*Oxford*, RICHARD WALKER,

*March* 3, 1814. *Surgeon and Apothecary.*

*On the Treatment of Pulmonary Consumption.*

I have witnessed the trial of a great variety of *specific remedies* in this disease, of which a great many cases presented themselves, and watched the effects of each to the last; until, by a constant repetition of failures, without one successful instance, I became wearied by continual disappointment, and sunk into a total indifference respecting the administration of others which followed.

Among these latter specifics I shall notice two only, viz. *digitalis* and *uva ursi*. Digitalis acted very evidently as a strong sedative, diminishing very perceptibly the rapidity of the circulation, apparent by the pulse becoming slower in consequence of its use; but the disease itself progressively advanced, and in the end terminated fatally. The only sensible alteration I observed in the different patients by its use, was an apparent abatement of the hectic fever, induced, as I concluded, by the debilitating powers of this deleterious drug, which I had no doubt hastened the end of those who took it, and in two instances *suddenly*, of which I was an eye witness. This is an effect in the exhibition of the digitalis which has been noticed, I believe, by others of the faculty as well as myself.

I observed nothing in the effect of uva ursi in pulmonary consumption to induce me, *in the least degree*, to alter my general opinion of the total inefficacy of *reputed specifics* in this disease.

At page 94 of a work entitled "Cases of Pulmonary Consumption, &c. treated with Uva Ursi," published in 1805, the author, in a very handsome manner, notices my attention to some of the cases which occurred at the Radcliffe Infirmary; and particularly in one case, where that gentleman states that he asked my opinion respecting the nature of the complaint, before he began the use of uva ursi, in which I concurred with him that it was evidently a case of *incipient pulmonary consumption*, and which opinion has since been confirmed by that person dying consumptive.

This gentleman likewise intimated a wish to know my opinion respecting the *efficacy* of uva ursi, in those cases which had passed under my observation, to which, influenced chiefly by his zeal, I had given very particular attention, especially one, whom I watched *incessantly*, from her entrance into the Infirmary to her death, a period of about seven seven weeks, viz. Mary Hopgood, a middle-aged woman, who, according to her account, had enjoyed very good health, until she had taken cold about six months before her admission into the Infirmary, which was in the beginning of July 1803; upon whom I could discover no sensible effect whatever, attributable to the uva ursi, except *nausea and loathing of nourishment*, when the medicine happened to be urged beyond what her stomach would bear, either upon the state of the pulse, or the progress of the disease; her urine, indeed, as is usual in the exhibition of uva ursi, was tinged with the color of that drug.

Thus circumstanced, and with the addition of further evidence of the same tendency, I found it impossible, consistently with my judgment and conscience, to give a favorable report, and therefore got rid of the question in the most delicate manner I could, being well aware that nothing but an equal degree of professional experience to my own, could have induced that gentleman to view this matter in the same light as I did myself.

Having been taught, by repeated observation, to consider uva ursi as an useless drug, I was induced to make the following trial respecting its supposed *sedative* quality: accordingly, I attended to the state of the pulse in two patients every night and morning for nearly six weeks, one of whom was taking uva ursi alone, and the other the same medicine, but in conjunction with opium; the result was, that in the latter person the pulse became progressively slower, but not in the former. In each instance I attended to the ratio of the pulse previously to their commencing this plan.

To the effect, therefore, of the opium which was joined with the uva ursi, as far as medicine was concerned, I ascribed, at that time, the alteration of the pulse, and amendment in the patient, who had the "disease in the urinary organs," which led the author to the trial of uva ursi in pulmonary consumption, and which is the subject of the first chapter.

This "elderly man" took, as in the author's words, "ten grains of uva ursi, rather more than that quantity of bark, and half a grain of opium, thrice in the day, for about seven weeks, and was then discharged, most remarkably improved in his general health, and very materially relieved with regard to the local affection." An alteration by no means attributable to the uva ursi, and which I am confident would have equally occurred had any other harmless substance been substituted in its stead, believing myself that *rest, and the comforts of the Infirmary*, had no small share in the beneficial effects which were produced on this poor old man, whom I well recollect, and who had before worked hard and fared but indifferently.

I should not have entered so minutely now on this subject, had I not thought it necessary, on account of the place to which I have assigned uva ursi in this work, and in which place of obscurity I should hope, in future, it will be quietly suffered to remain.

Thus it happens that inefficacious drugs obtain a place in the catalogue of remedies, and unfortunately retain their place, because contemporary or succeeding practitioners have wanted a due degree of experience to fortify them with a sufficient degree of confidence to put a negative upon them! for I am certain, had this gentleman witnessed the *whole practice of an Infirmary*, with his discernment and attention, for twenty years, he could no more have proposed uva ursi as a remedy for pulmonary consumption, in any stage of it, than he could have proposed it for the purpose of arresting or checking the progress of advancing old age.

Upon this occasion, according to the author's directions, the healthy-looking or well-preserved leaves only were used, (the stalks and withered leaves being rejected) which amounted, upon an average, to somewhat less than three-fourths, by weight, of the whole of the specimens, as received from the druggist.

There are two evils arising from a pertinacious adherence to a *specific*, mistakenly so considered. 1st. All other means, consisting of an appropriate regimen, palliating medicines, and various other salutary measures, are entirely overlooked; whereas, by a judicious application of these, nature might be enabled in many cases, particularly in recent affections, to conquer the disease, and in all cases the patient's sufferings mitigated to the last. 2d. It too commonly happens that the fallacious specific is not entirely of a passive nature: hence the sufferings of the patient are aggravated by the supposed remedy. This accumulation of evil was most strikingly exemplified in the instance of *Mary Hopgood* above-mentioned; and which, to any person whose long experience and great familiarity with cases of a similar nature had taught real or true professional wisdom, must necessarily prove extremely irksome and distressing.*

* The Radcliffe Infirmary is conducted upon the most liberal plan, respecting the accommodation of the faculty and the patients. In this instance the patient, Mary Hopgood, in order to be free from the annoyances incident to a large ward, was placed in a smaller one, (a comfortable airy room,) where she possessed every advantage to be obtained in a private house.

I should not, for various reasons, have expressed my sentiments so freely on this work, but for the great importance of the subject, together with the consideration that my experience of the effects of medicine on diseases generally, conjointly with my observations in this particular instance, renders it morally impossible I can err when I assert, that *the administration of this drug in* PULMONARY CONSUMPTION *aggravates the sufferings naturally incident to the disease itself*, unless confined to so small a dose as to render it *merely inert.*

I wish it to be understood that I do not take upon myself to assert positively that uva ursi has no effect with respect to *altering the ratio of the pulse*, provided it be exhibited in doses sufficiently large; but as I have observed before, respecting the exhibition of digitalis, I consider this as no criterion of its salutary effects on the disease, but rather otherwise. It is remarkable, however, that in another instance of its exhibition for five or six weeks as before, the author and myself attended separately to the state of the pulse, and at the end of that time, upon comparing notes, it was found there had been no sensible alteration in the pulse attributable to the uva ursi.

With respect to the astringent quality of uva ursi, and its peculiar determination to the urinary passages, demonstrated by its effect on the urine of some patients, when persisted in for a length of time, which I presume originally led to the exhibition of it in various diseases of the urinary organs, and for which it still preserves *a questionable kind of celebrity*; as in catarrhus vesicæ, diabetes, &c. I confess I could never observe any decidedly good effects from its exhibition in such disorders, and I am certain, as I shall have occasion to speak of more fully hereafter, that much more may be done by other means, particularly in the former of these diseases.

Several professional persons, as I well know, entertain an opinion that at the worst uva ursi is an inoffensive medicine, believing that "if it does no good, it can do no harm." In order, however, to satisfy myself respecting the peculiarly unpleasant sensation this drug produces in the stomach, so frequently described to me by patients, as well since I left the Infirmary as before, when prepared as above, I took twenty grains of it myself about two hours after breakfast, which deranged my stomach so much as entirely to unfit it for relishing my dinner; and, by the bye, my stomach is not easily diverted from its purpose when dinner presents itself. This effect is commonly produced in the stomach of persons

 debilitated debilitated by disease, when so much as ten grains are taken, or from thence to fifteen.

Strictly speaking, in my opinion there is no specific medicine for any internal disease to which human nature is liable. Syphilis, which might be considered as a contradiction to this assertion, arises, in all instances within our knowledge, from infection, and may therefore be considered as the effect of a poison of a peculiar nature, consequently differing entirely from diseases, properly so called, and for which poison mercury is the antidote.*

If this be the fact, as experience and reason have convinced me, how extremely injurious, as well as futile, are the constant attempts to discover specific medicines, and to introduce them to the credulous community!

Whether subsequent experience has in any way affected the opinion of the author respecting *the efficacy of uva ursi in pulmonary consumption*, since his publication, I know not; mine is still the same, and I am confident must ever remain so.

(To be continued.)

* I shall have occasion hereafter to make a distinction, as I consider it, between *actual diseases* and *mere indispositions*: pulmonary consumption I consider as coming under the former class or denomination, and ague, or intermittent fever, under the latter.

N.B.—In cases where an astringent is indicated of the absorbent tonic kind, which certainly is not in the earlier stage of pulmonary consumption, I think liquor calcis taken in the usual way with milk, or, according to circumstances, in combination with cinchona, promises to be most efficacious.

